# Proteomics and image screening data of cellular secretomes and their biological effects: Comparing the signals sent by cardiac stromal cells and dermal fibroblasts in culture

**DOI:** 10.1016/j.dib.2022.107963

**Published:** 2022-02-16

**Authors:** Anny Waloski Robert, Michel Batista, Jhonatan Basso Lino, Marco Augusto Stimamiglio, Alessandra Melo de Aguiar

**Affiliations:** a Stem Cells Basic Biology Laboratory, Carlos Chagas Institute – FIOCRUZ/PR, Curitiba, Parana 81350-010, Brazil; b Mass Spectrometry Facility RPT02H, Carlos Chagas Institute – FIOCRUZ/PR, Curitiba, Parana 81350-010, Brazil

**Keywords:** Secretome, Conditioned medium, Differentiation, Proteomics, High-content screening, Mass spectrometry, LC-MS/MS

## Abstract

The study of the secretome of different cell types has gained prominence over the years due to its role in understanding the cell microenvironment and possible uses in acellular therapies. Approaches in this field include proteomic characterizations of the secretomes as well as evaluating their potential to induce cell and tissue responses. Here, we present the mass spectrometry proteomics data from a characterization of the secretome of cardiac resident stromal cells (CRSCs) and dermal fibroblasts in order to compare their compositions. To evaluate the potential for cell proliferation, differentiation, migration, and adhesion, *in vitro* assays were performed and analyzed using a high-content imaging system. For each assay, specific analysis strategies were developed to quantify the generated data. These datasets provide insights into the differences and similarities between secretomes from different cell sources. It also describes methodologies for analyzing images from different *in vitro* assays using high-throughput automated imaging.

## Specifications Table

**Table:** 

Subject	Biological sciences
Specific subject area	Cell niche, Cell biology and differentiation, Proteomic characterization.
Type of data	Table, Figure
How data were acquired	High-content screening and analysis system: Operetta CLS™ High-Content Analysis System and Harmony version 4.5 and 4.8 software, PerkinElmer; Mass spectrometry: Easy-nLC 1000 coupled to LTQ Orbitrap XL ETD, MaxQuant version 1.6.1.0;
Data format	Raw Analyzed Filtered
Parameters for data collection	Label-free mass spectrometry (LC-MS/MS) analysis was performed with concentrated conditioned medium (CM) derived from: (a) ventricle-derived cardiac resident stromal cells (vCRSC) from three human donors; (b) human dermal fibroblasts (HDF, commercial primary cultures) from three cell culture replicates; and (c) non-conditioned medium (*n* = 2). The peptides were analyzed in triplicate (vCM3 and nCM1) or duplicate (vCM1,2, fCM1, 2, 3 and nCM2). The MS data were acquired in DDA mode (MS1 full scan performed in the orbitrap and the MS2 in the linear trap quadrupole). Immunostaining evaluation was performed in the Operetta CLS™ High-Content Analysis System, using a 20x objective to capture 30 to 49 images for each well, depending on the assay. The channels used were: brightfield, digital phase contrast, DAPI and Alexa 488. After acquisition, images were analyzed in Harmony software to quantify the number of objects (e.g., nuclei or ki67+ cells) or the area (e.g., open area or Alexa 488+ area).
Description of data collection	vCRSC from three human donors and HDF were cultured to collect CM. Fresh media, termed non-conditioned medium (nCM) was used as a control for all experiments. CM and nCM were concentrated, and the equivalent of 30 µg of protein was used for mass spectrometry analysis. Functional assays were performed with non-concentrated CM. H9c2 cells were cultured over 7 and 15 days with CMs and nCM to evaluate its potential in stimulate cell proliferation (assessed by DAPI/Ki67 immunostaining) and cardiac differentiation (measured based on the cardiac troponin I immunostained area). To evaluate the potential of CM in promote cell migration, a scratch assay was performed. After the scratch in cell monolayer, H9c2 cells and HUVEC were treated with CMs and nCM and a 24 h time lapse was performed to quantify the closed area over time. In addition, the number of H9c2 cells and HUVEC adhered in cell plates after treatment with CMs and nCM over 10 min, 20 min and 40 min was also verified.
Data source location	Institution: Carlos Chagas Institute – FIOCRUZ/PR City/Town/Region: Curitiba, Parana Country: Brazil
Data accessibility	The mass spectrometry proteomics data have been deposited to the ProteomeXchange Consortium via the PRIDE partner repository with the dataset identifier PXD026451 (https://www.ebi.ac.uk/pride/archive/projects/PXD026451). Images and analysis derived from Operetta CLS™ High-Content Analysis System were available in Mendeley Data Repository, doi: 10.17632/72vcr9c6rk.1 (http://dx.doi.org/10.17632/72vcr9c6rk.1)
Related research article	J.B Lino, A.W. Robert, M.A. Stimamiglio, A.M de Aguiar, Comparative analysis of the potential of the secretomes of cardiac resident stromal cells and fibroblasts, IUBMB Life. (2021) 1–11. https://doi.org/10.1002/iub.2557

## Value of the Data

• The data provide a proteomics characterization of human ventricle-derived cardiac resident stromal cells (vCRSCs) and dermal fibroblast secretomes, as well as an evaluation of their ability to influence cellular behaviors, which can be useful in identifying the best source of cells for soluble factors to be used in potential future acellular therapies.
• The data can be useful for other groups working on secretome characterization. In addition, the strategy of analyzing data obtained from a high-content screening system is a tool that can be used in a wide variety of studies.
• Further analysis and additional assays can be performed, allowing comparison with other secretomes, both in terms of protein composition and functionality.
• A detailed description of the analysis method for images obtained from a high-content screening system can be used for efficiently evaluating cardiac differentiation, cell proliferation, and migration.

## 1 Data Description

**Fig. 1:**
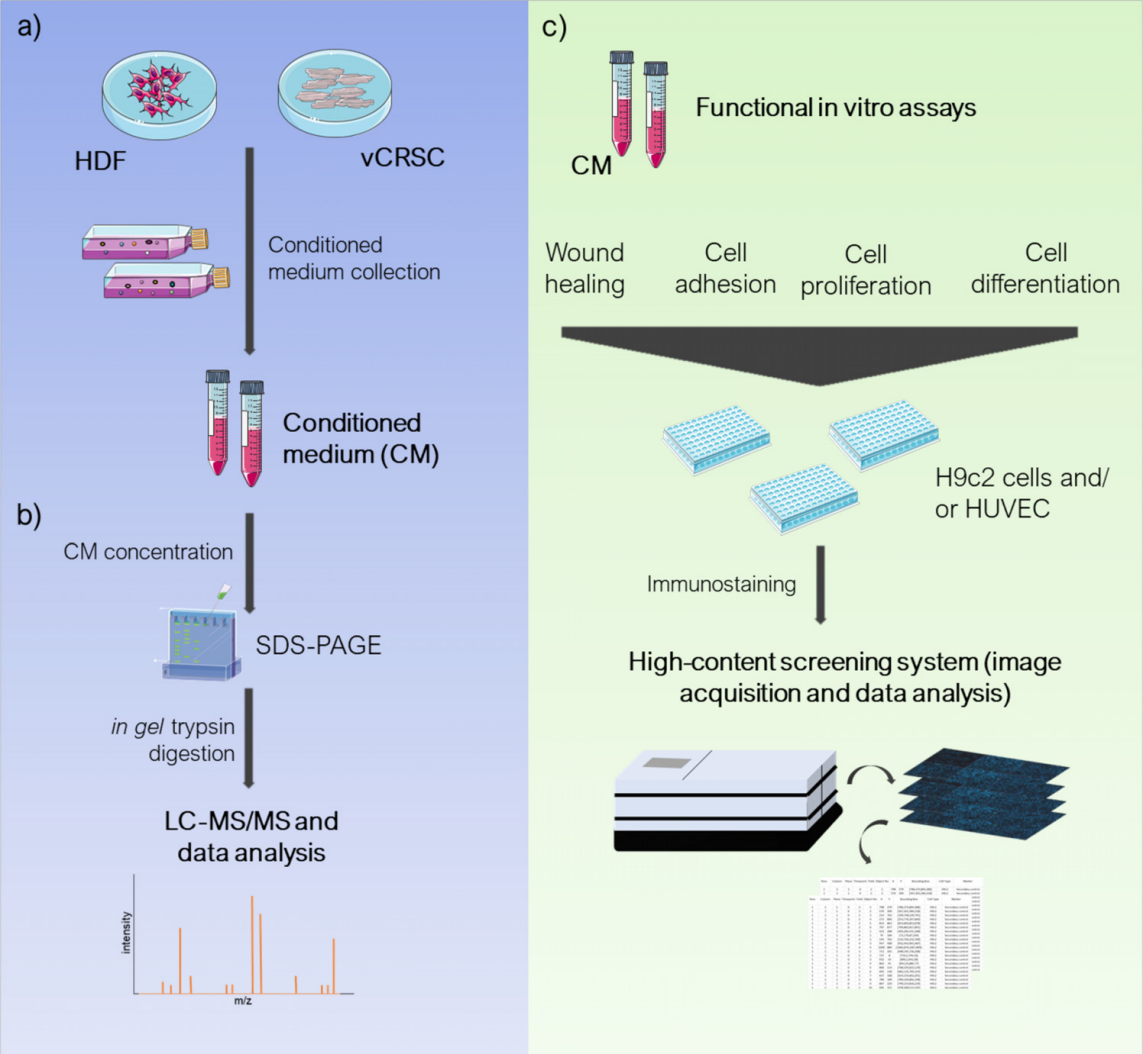
Data collection workflow. (a) Conditioned medium (CM) were collected from human ventricle-derived cardiac resident stromal cells (vCRSC) and human dermal fibroblasts (HDF). Non-conditioned medium was also collected in parallel as control. (b) Samples were concentrated, and in-gel (SDS-PAGE) trypsin digested for mass spectrometry (LC-MS/MS) data collection and analysis. (c) Additionally, CM samples were assayed for functional cell responses *in vitro*. H9c2 cells or HUVECs were used to assess the potential of CM to modulate cell adhesion, proliferation, differentiation and wound healing. Cell data were collected with a high-content imaging system. The images were adapted from Servier Medical Art (http://smart.servier.com/), licensed under a Creative Commons Attribution 3.0 Unported License (https://creativecommons.org/licenses/by/3.0/).

The dataset presented here contains the raw proteomic data from conditioned medium (CM) obtained from human ventricle-derived cardiac resident stromal cells (vCRSC) from three donors as well as from normal neonatal human dermal fibroblasts (NHDF-neo) from three independent cell cultures [1,2]. In addition, we showed the analysis of high-content screening system data obtained from cell proliferation, differentiation, adhesion, and migration assays. The schematic design of experiments is depicted in Fig. 1.

The proteomic raw data of the vCRSC-derived CM from donor 1 (vCM1), donor 2 (vCM2), and donor 3 (vCM3), the CM derived from dermal fibroblasts (fCM1, 2, and 3) and the non-conditioned medium (nCM1 and 2) as well as the proteomic analysis from MaxQuant are available in PRIDE repository under accession number PXD026451 [3]. More details can be found in Table 1.

**Table 1:** Description of files deposited in the ProteomeXchange Consortium via the PRIDE partner repository (dataset identifier PXD026451).

File name	Content/ Description
20160513_vCM1_1.RAW	raw data from vCM from donor 1 - 1
20160513_vCM1_2.RAW	raw data from vCM from donor 1 - 2
20160513_vCM2_1.RAW	raw data from vCM from donor 2 - 1
20160513_vCM2_2.RAW	raw data from vCM from donor 2 - 2
20141205_vCM3_1.RAW	raw data from vCM from donor 3 - 1
20141205_vCM3_2.RAW	raw data from vCM from donor 3 - 2
20141205_vCM3_3.RAW	raw data from vCM from donor 3 - 3
20160513_fCM1_1.RAW	raw data from fCM - Replicate 1 - 1
20160513_fCM1_2.RAW	raw data from fCM - Replicate 1 - 2
20180806_fCM2_1.RAW	raw data from fCM - Replicate 2 - 1
20180806_fCM2_2.RAW	raw data from fCM - Replicate 2 - 2
20180806_fCM3_1.RAW	raw data from fCM - Replicate 3 - 1
20180806_fCM3_2.RAW	raw data from fCM - Replicate 3 - 2
20141205_nCM1_1.RAW	raw data from nCM - Replicate 1 - 1
20141205_nCM1_2.RAW	raw data from nCM - Replicate 1 - 2
20141205_nCM1_3.RAW	raw data from nCM - Replicate 1 - 3
20160513_nCM2_1.RAW	raw data from nCM - Replicate 2 - 1
20160513_nCM2_2.RAW	raw data from nCM - Replicate 2 - 2
Hsapiens_uniprot_71599entries_17052018.fasta	fasta file from proteome database used for analysis
txt.zip	MaxQuant analysis data

We evaluated CMs *in vitro* potential to interfere with cell processes: we performed cell proliferation, differentiation, adhesion, and migration assays in H9c2 rat cardiomyoblasts and human umbilical vein endothelial cells (HUVEC). The analysis of the cell phenotypes was addressed by the Operetta CLS™ high-content analysis system and the Harmony software (PerkinElmer).

Proliferation and cardiac differentiation assays were performed with H9c2 cells that were kept in the CMs for seven and 15 days. Cellular proliferation was quantified based on the Ki67 staining presented in the nuclei of cells. Cardiac differentiation was confirmed by staining cells with the anti-cardiac troponin I (cTnI) antibody and quantifying the stained area. Using an *in vitro* wound healing assay, H9c2 cells and HUVEC were treated with CMs and monitored over 24 h, with images acquired every 6 h, to evaluate the change of the open area. Finally, the cell adhesion capacity was verified at early H9c2 and HUVEC culture times: 10, 20, and 40 min after plating with CMs.

The data generated after image analysis with Harmony v.4.5 or 4.8 software (PerkinElmer), as well as the images of the analyzed plates (one for each time and for each experiment) and a file indicating the treatments/ well and the wells that were excluded from the analysis are available at Mendeley Data Repository (doi: 10.17632/72vcr9c6rk.1) [4]. These data are separated into folders, one for each assay. Table 2 highlights information about the *in vitro* functional assays, as the controls used, and presents a brief description of files deposited in Mendeley Data Repository. More details of the exclusion criteria and the analysis methodology in Harmony software (PerkinElmer) were described in the Experimental Design, Materials and Methods section.

**Table 2:** Description of data files deposited in Mendeley Data Repository.

	Wound healing	Adhesion	Proliferation	Differentiation
Cell type	H9c2, HUVEC	H9c2, HUVEC	H9c2	H9c2

Experimental controls	EBM-2 complete (positive control for HUVEC); DMEM 10%FBS (positive control for H9c2)	EBM-2 complete and with no supplements as positive and negative control for HUVEC, respectively; DMEM 10%FBS or 1% FBS as positive and negative control for H9c2, respectively	DMEM 10%FBS (positive control); DMEM 1% (negative control)	DMEM 10%FBS (negative control); DMEM 1% (positive control)

Stainings	–	DAPI	DAPI, anti-Ki67 antibody, secondary antibody control	DAPI, anti-cardiac troponin I antibody, secondary antibody control

Image analysis (global or individual)	Global	Individual	Individual	Global

Description of folder content	- Representative images from the whole plate over time (0, 6, 12, 18, 24 h). - XLSX file: Results for HUVEC and H9c2 cells, containing the open area over time (Timepoints: 0 = 0 h; 1 = 6 h; 2 = 12 h; 3 = 18 h; 4 = 24 h). - File containing well identification and those considered for analysis.	- Representative images from the whole plate for each cell type and for each timepoint (10, 20 or 40 min). - XLSX files: Results were separated for cell type, containing the total number of objects (nuclei) identified/ time. - File containing well identification.	- Representative images from the whole plates, for each timepoint (7 or 15 days), each experiment and for each image channel evaluated. - XLSX files: Results by object population (nuclei): for each identified nucleus (object number) it was verified if there was Ki67 staining (0 = absent; 1 = present). - File containing well identification and those considered for analysis.	- Representative images from the whole plate for each timepoint (7 or 15 days) and for each image channel evaluated. - XLSX files: Results were separated for analyzed timepoint, containing the measure of Alexa488 + area and total number of objects (nuclei) identified. - File containing well identification and those considered for analysis.

## 2 Experimental Design, Materials and Methods

### 2.1 Cell culture and conditioned medium collection

This study was approved by the ethics committee of the 10.13039/501100006507Oswaldo Cruz Foundation (CAAE number: 48374715.8.0000.5248). Ventricle-derived cardiac resident stromal cells (vCRSC) were isolated with culture conditions following a previously established methodology [5]. Table 3 shows the characteristics of the vCRSC donors. The cells were maintained on type I collagen-coated plates in Dulbecco's MegaCell® supplemented with 5% fetal bovine serum (FBS), 0.1 mM β-mercaptoethanol (BME), 1% non-essential amino acids (NEAA), 2 mM L-glutamine, 100 IU/mL penicillin, 0.1 mg/mL streptomycin, and 5 ng/mL of basic fibroblast growth factor (bFGF). Normal neonatal human dermal fibroblasts (NHDF-neo, referred to as HDF in this paper, Lonza® cat.: CC-2509) and H9c2 cells (ATCC®, cat.: CRL‐1446™) were kept in Dulbecco's Modified Eagle Medium (DMEM) 10% FBS, 1% L-glutamine, 100 IU/mL penicillin, and 0.1 mg/mL streptomycin. Human umbilical vein endothelial cells (HUVEC, Lonza® cat.: C2519A) were kept in endothelial cell growth medium‐2 (EBM-2™, Lonza) supplemented with 5% FBS, human FGF, human epidermal growth factor (EGF), human vascular-endothelial growth factor (VEGF), insulin‐like growth factor 1 (R3‐IGF‐1), ascorbic acid, hydrocortisone, and GA‐1000 (30 mg/mL gentamicin and 15 μg/mL amphotericin). All cell types were kept in a humidified incubator at 37 °C and 5% CO_2_. For collecting the conditioned medium (CM), the HDFs were initially adapted to the same culture conditions as vCRSC: in type I collagen-coated plates using Dulbecco's MegaCell® supplemented with 5% FBS, 0.1 mM BME, 1% NEAA, 2 mM L-glutamine, 100 IU/mL penicillin, 0.1 mg/mL streptomycin, and 5 ng/mL bFGF.

**Table 3:** Information on tissue donors used in this study. WIT: warm ischemia time; CIT: cold ischemia time.

Donor	Age	Gender	WIT (hours)	CIT (hours)
vCRSC1	33 years	Female	2:00	10:20
vCRSC2	30 years	Male	2:10	27:00
vCRSC3	27 years	Male	1:40	24:45

**Fig. 2:**
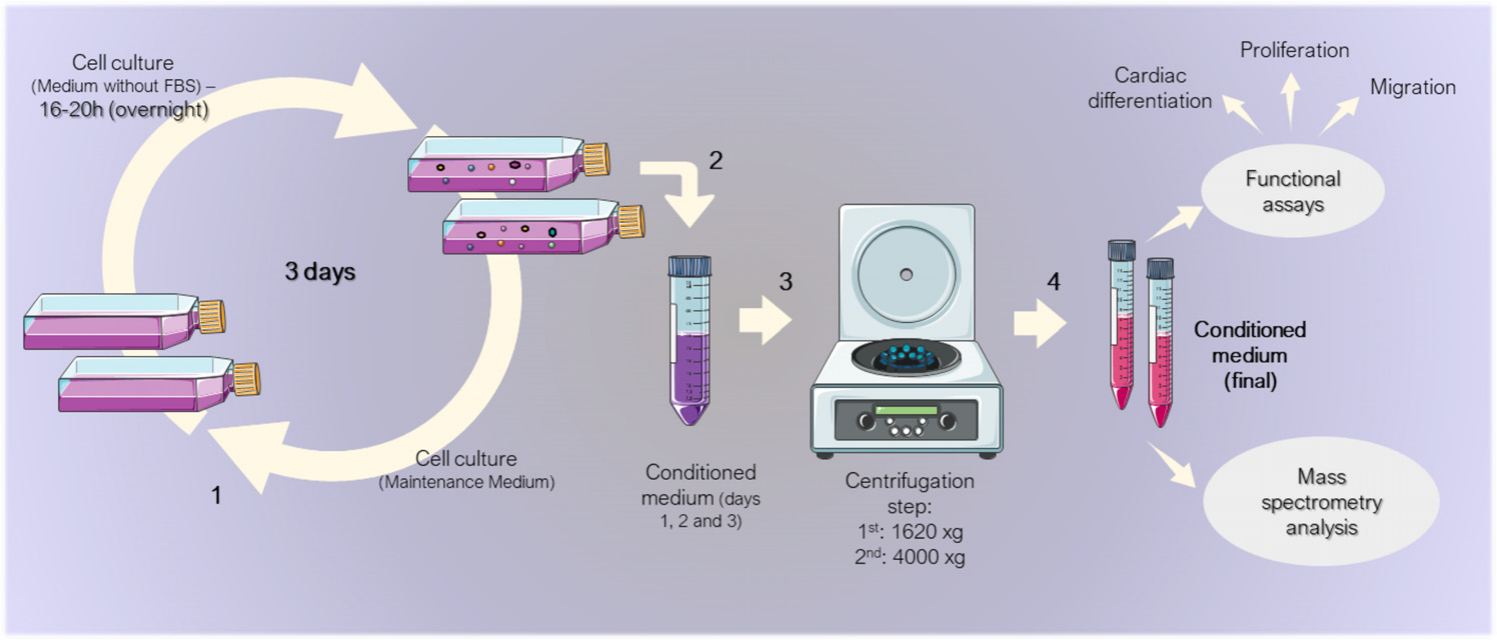
**Schematic representation of CM collection.** (1) After reaching 80% of confluence, cell cultures were incubated with medium without FBS overnight. The conditioned medium was then collected (2), and cell cultures returned to the maintenance medium during the day, with the process starting again at the end of the day. This was performed over three days. (3) The CMs collected were centrifuged twice and stored at 4–8 °C until the end of collections. (4) The CMs collected over three days were pooled and stored at −80 °C until used in mass spectrometry analysis or functional assays. The images were adapted from Servier Medical Art (http://smart.servier.com/), licensed under a Creative Commons Attribution 3.0 Unported License (https://creativecommons.org/licenses/by/3.0/).

Upon reaching 80% confluence, the vCRSC and HDF cultures were washed three times with 1x phosphate buffer saline (PBS), following which Dulbecco's MegaCell® supplemented only with 0.1 mM BME, 1% NEAA, 2 mM L-glutamine, and 100 IU/mL penicillin, and 0.1 mg/mL streptomycin was added to culture flasks. This medium remained in contact with the cells for 16–20 h (overnight). Subsequently, the CM was collected, and the cells were again incubated with complete Dulbecco's MegaCell® medium (with FBS and bFGF) for 6–8 h. The process of PBS washing and adding medium without FBS and bFGF was then repeated. This was done for a total of three days (three consecutive CM collections). On each day, the collected medium was centrifuged: first at 1620 xg for 5 min and then at 4000 xg for 20 min, both at 4–8 °C. The CM of each of the three days was pooled and then stored at −80 °C until use. This was done for each of the three vCRSC donors and HDF replicates. In parallel, the non-conditioned medium (nCM) was also stored (Dulbecco's MegaCell® supplemented only with 0.1 mM BME, 1% NEAA, 2 mM L-glutamine, and 100 IU/mL penicillin, 0.1 mg/mL streptomycin). Fig. 2 summarizes this process.

For LC-MS/MS analysis, 15 mL of each CM was concentrated using Amicon® Ultra 15 mL Centrifugal Filters of 10,000 NMWL (Merck, cat.: UFC901024), followed by protein quantification using a Qubit™ Protein Assay Kit (Invitrogen™, cat.: Q33211). For functional assays, the CM was used pure, supplemented only with 100 IU/mL penicillin, 0.1 mg/mL streptomycin, and 2 mM L-glutamine.

### 2.2 LC-MS/MS

Thirty micrograms of protein from each sample were mixed with 4x SDS-PAGE sample buffer (160 mM Tris–HCl pH 6.8, 4% SDS, 10% b-mercaptoethanol, 24% glycerol, and 0.02% bromophenol blue) to final buffer concentration of 1x and were resolved in 10% SDS-PAGE. Then, SDS-PAGE lanes were sliced and underwent *in gel* trypsin digestion. The samples were analyzed at the mass spectrometry facility RPT02H/Carlos Chagas Institute - Fiocruz Paraná. The peptides were analyzed in triplicate (for vCM3 and nCM1 samples) and in duplicate (for other samples) by LC-MS/MS in an Easy-nLC 1000 online with a LTQ Orbitrap XL ETD (Thermo Scientific). The chromatography was performed in a C18 column (30 cm length, 75 µm I.D., 1.9 µm particle) with a flow of 250 nL/min and a linear gradient of 5–40% acetonitrile in 0.1% formic acid and 5% DMSO for 2 h. The MS data were acquired in DDA mode, with the MS1 full scan performed in the orbitrap (60,000 resolution) and the MS2 in the linear trap quadrupole, where the top 10 most intense ions were subjected to CID fragmentation. Three consecutive run batches, spaced in time, were carried out using the same experimental protocol and equipment, according to the following scheme: vCM3 and nCM1 in parallel; vCM1, vCM2, fCM1, and nCM2 in parallel; fCM2 and fCM3 in parallel.

### 2.3 Data analysis

The raw data from LC-MS/MS (from all the samples) were analyzed in MaxQuant software, version 1.6.1.0 [6,7]. The default parameters were used, including trypsin as protease, carbamidomethylation of cysteine as fixed modification, acetylation of protein N-terminal, and methionine oxidation as variable modification. The *Homo sapiens* database containing 71,599 entries was used, downloaded from Uniprot on May 17, 2018. Additional information about the analysis parameters can be found in the PRIDE repository (PXD026451).

### 2.4 Immunofluorescence

The cell cultures were washed once with PBS and then fixed with 4% paraformaldehyde. Cells were subsequently permeabilized with 0.5% Triton X-100 for 30 min, blocked with 1% bovine serum albumin (PBS/BSA 1%) for 1 h, and incubated with primary antibody (specific for each assay) for 1 h at room temperature or overnight at 4 °C. Next, cells were washed with PBS, incubated with Alexa Fluor® 488 Goat Anti-Rabbit (IgG) secondary antibody for 1 h and, after PBS washes, 4′,6-diamidino-2-phenylindole (DAPI) was added for 10 min to stain the nuclei.

Image acquisition was performed in Operetta CLS™ high-content analysis system (PerkinElmer). Image analysis was carried out with the Harmony software (PerkinElmer). The images from all the functional assays were obtained with a 20x Air, NA 0.4 objective, in non-confocal mode, binning = 2, with laser power ranging from 20% (Brightfield) to 50–75% (fluorescent channels), with exposure time and focus adjusted for each assay. Table 4 summarizes the experiment design and the image channels used for imaging acquisition for each assay.

**Table 4:** Experimental design for the *in vitro* assays and configuration for image acquisition in Operetta CLS™ high-content analysis system (Perkin Elmer).

	Assay	Wound healing	Adhesion	Proliferation	Differentiation
Experiment design	Well size	96-well plate	24-well plate	24-well plate	96-well plate
	Number of images/ well	30	49	49	36

Image channels	Brightfield (Exc: transmitted light; Emi: 655–760 nm)	X	–	–	–
	Digital phase contrast (Exc: transmitted light; Emi: 655–760 nm)	X	–	–	–
	DAPI (Exc:355–385 nm; Emi: 430–500 nm)	–	X	X	X
	Alexa 488 (Exc: 460–490 nm; Emi: 500–550 nm)	–	–	X	X

Exc: Excitation; Emi: Emission.

**Fig. 3:**
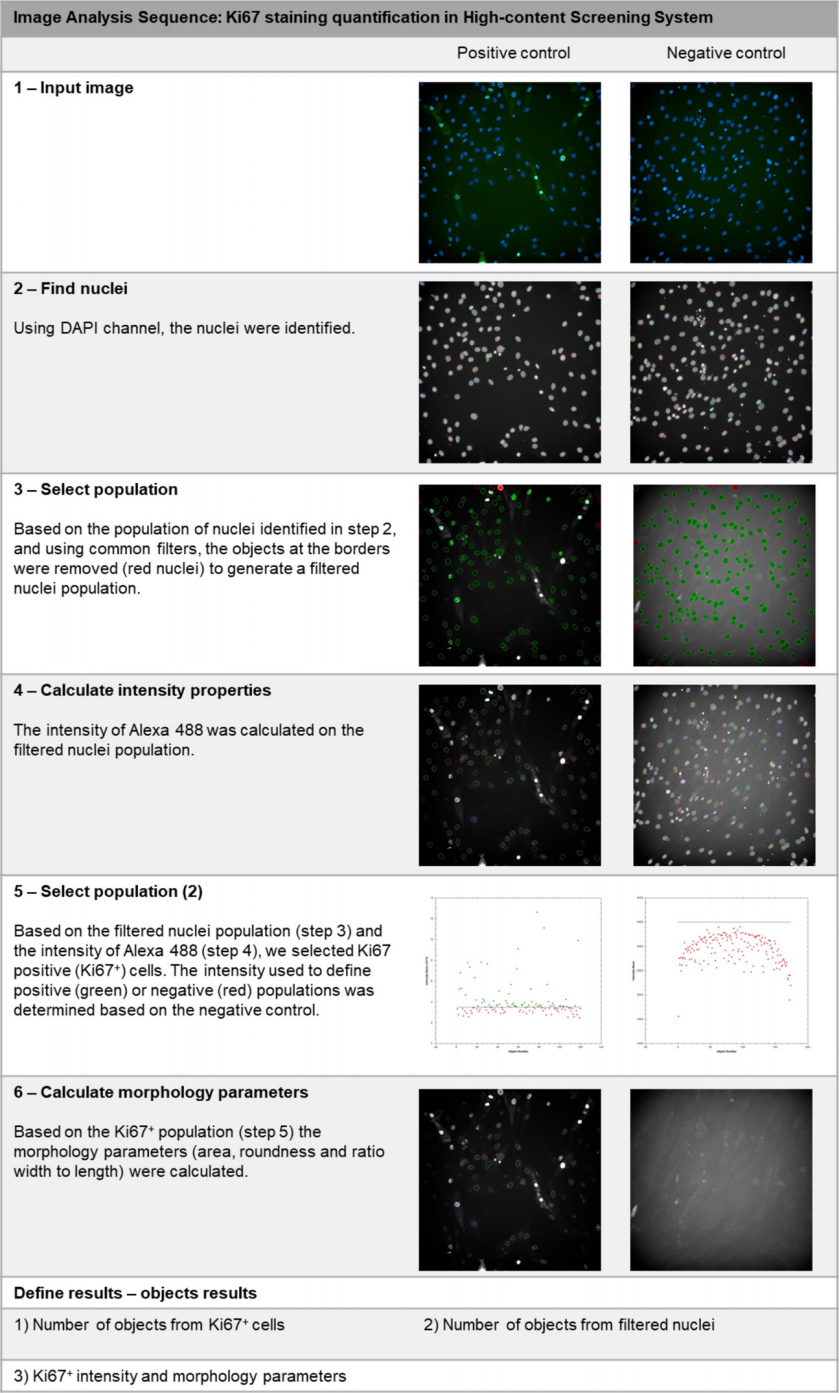
Image analysis sequence for quantification of Ki67 staining.

**Fig. 4:**
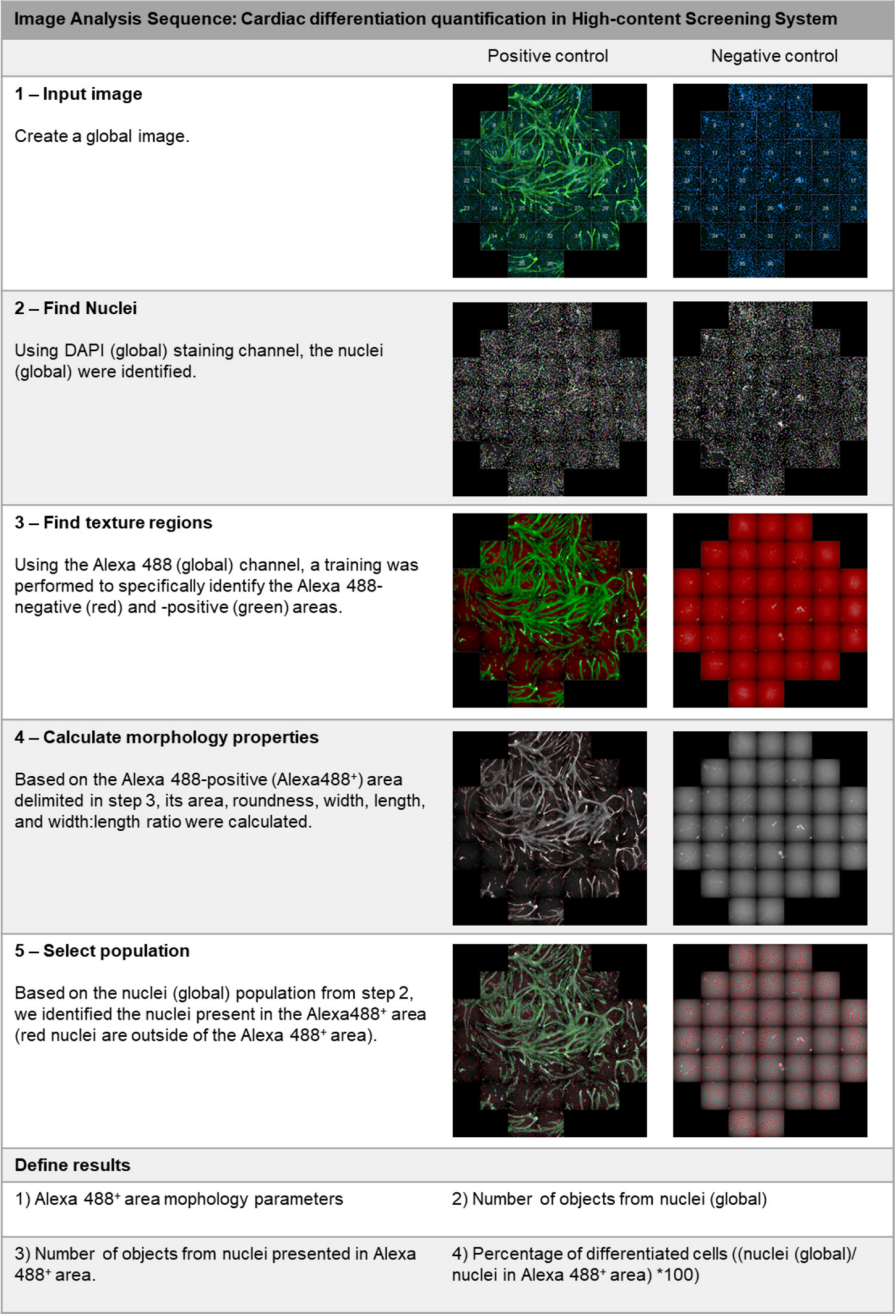
Image analysis sequence to quantify cardiac differentiation efficiency.

**Fig. 5:**
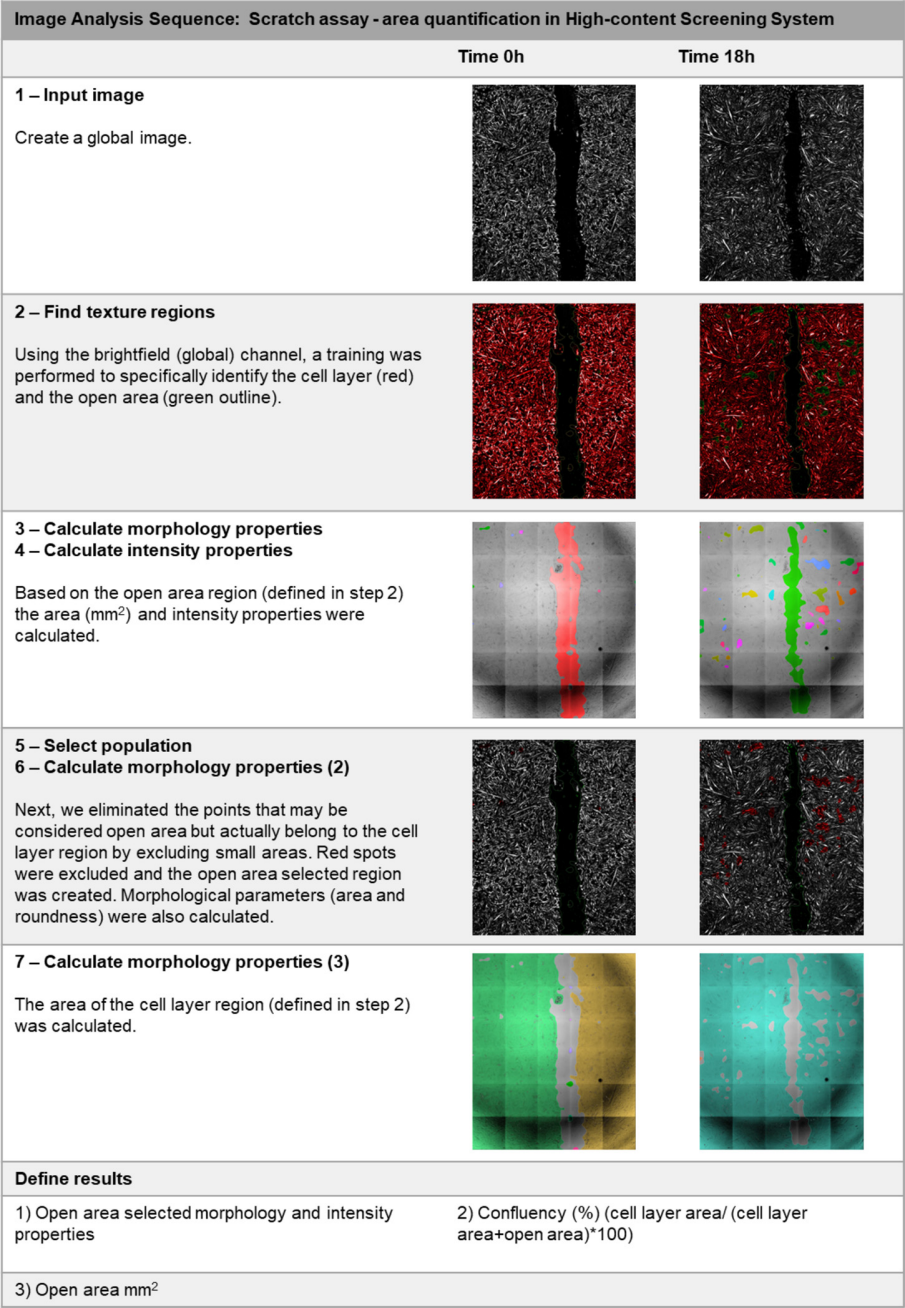
Image analysis sequence for *in vitro* wound healing assay analysis.

### 2.5 Proliferation and cardiac differentiation analysis

For the proliferation and cardiac differentiation assays, H9c2 cells were cultured with DMEM 10%FBS, DMEM 1%FBS as controls, as well as with vCM1, vCM2, vCM3, fCM, or nCM for seven and 15 days, following which the cells were fixed and immunostained.

For the proliferation assay, 1.2 × 10^4^ cells were plated in each well of 24-well plates. The treatments started 24 h after cell plating. The proliferating cells were identified by immunostaining for nuclear protein Ki67 using an anti-Ki67 antibody (Abcam, cat.: ab15580, dilution 1:300) and the Alexa Fluor® 488 Goat Anti-Rabbit (IgG) secondary antibody in association with nuclear counterstain DAPI. Forty-nine photos were acquired in each well and analyzed individually. Fig. 3 details the image analysis sequence used to quantify the total number of cells and the number of Ki67^+^ cells in the cultures treated with CM and nCM. Briefly, DAPI staining was used to distinguish the nuclei from cells. Next, using the images obtained from the Alexa 488 channel, we determined the number of cells that had nuclear staining for Ki67 (Ki67^+^ cells). The analysis from [1], depicted in Fig. 2, was generated based on a quantification of the total number of nuclei and the Ki67^+^ nuclei from 23 out of 49 photos.

To assess cardiac differentiation, 2.5 × 10^3^ cells were plated in each well in 96-well plates and with treatments starting after 24 h. The cardiomyocytes presented in the H9c2 cell culture after CM treatment were characterized using the anti-cardiac troponin I antibody (Santa Cruz, cat.: sc-15368, dilution 1:100) and the Alexa Fluor® 488 Goat Anti-Rabbit secondary antibody. Thirty-six photos were acquired in each well and, using Harmony 4.8 software, they were combined to form only one image for each well (global image). Fig. 4 describes the image analysis procedure for the images generated by the Operetta CLS™ high-content analysis system (PerkinElmer) to determine cardiac differentiation efficiency. Briefly, we trained the Harmony 4.8 software to identify the texture of differentiated cells (Alexa 488/ cTnI^+^) from non-differentiated ones (Alexa 488/ cTnI^−^). After that, the stained area (cTnI^+^) and total nuclei were quantified. The wells that had problems at some stage of immunofluorescence or in the reading on Operetta CLS™ high-content analysis system (PerkinElmer) were excluded from the analysis.

### 2.6 Adhesion and migration analysis

A description of cell plating density and culture times can be found in [1]. In the adhesion assay, after DAPI staining, 49 photos were taken in Operetta CLS™ high-content analysis system (PerkinElmer) for each well and time. The analysis was performed in Harmony 4.5 software (PerkinElmer) and consisted of the identification and quantification of nuclei. The treatments performed for HUVEC cultures were: vCM1, vCM2, vCM3, fCM, nCM, EBM2 complete medium (positive control) and EBM2 without supplements (negative control). The treatments performed for H9c2 cell cultures were: vCM1, vCM2, vCM3, fCM, nCM, DMEM 10% (positive control) and DMEM 1% (negative control).

For wound healing assay, a time-lapse analysis was carried out with Operetta CLS™ high-content analysis system (PerkinElmer). Before starting the assay, the equipment was set for the standard cell culture condition: 37 °C and 5% CO_2_. The wells were photographed every 6 h over a 24 h period. To cover the entire scratch, 30 photos were acquired with a 20x objective and were combined to form a single image from the whole well (global image) (Fig. 5). Then, using Harmony 4.8 software (PerkinElmer), we measured the open area each time (Fig. 5). Wells that did not exhibit valid values (NaN) at any of the intervals were excluded from the analysis, as were wells in which the initial scratch (time 0 h) was improperly done or outside of the image, or where the percentage reduction in area was negative at more than one point in time. The treatments performed for HUVEC cultures were: vCM1, vCM2, vCM3, fCM, nCM, and EBM2 complete medium (control). The treatments performed for H9c2 cell cultures were: vCM1, vCM2, vCM3, fCM, nCM, and DMEM 10%FBS (control).

## Ethics Statements

This study was conducted in accordance with The Code of Ethics of the World Medical Association (Declaration of Helsinki). The research was approved by the ethics committee of the 10.13039/501100006507Oswaldo Cruz Foundation (CAAE number: 48374715.8.0000.5248).

## CRediT authorship contribution statement

**Anny Waloski Robert:** Conceptualization, Writing – original draft, Writing – review & editing, Visualization. **Michel Batista:** Writing – original draft, Data curation. **Jhonatan Basso Lino:** Investigation. **Marco Augusto Stimamiglio:** Writing – review & editing, Funding acquisition. **Alessandra Melo de Aguiar:** Writing – review & editing, Funding acquisition, Project administration.

## Declaration of Competing Interest

The authors declare that they have no known competing financial interests or personal relationships that could have appeared to influence the work reported in this paper.
